# Dysphagia and thickened esophageal wall: the application of ultra-slim gastroscope in the diagnosis and treatment of phlegmonous esophagitis

**DOI:** 10.1055/a-2587-9014

**Published:** 2025-05-09

**Authors:** Guangzhao Li, Huikai Li, Xiuxue Feng, Fen Deng

**Affiliations:** 1 Department of Gastroenterology, The First Medical Center of PLA General Hospital, Beijing, China; 2 Department of Gastroenterology, The 969th Hospital of the PLA Joint Logistics Support Force, Hohhot, China

A 61-year-old woman presented with persistent sore throat for 17 days and progressively worsening dysphagia for 13 days. Computed tomography (CT) of the chest showed a thickened wall of the entire esophagus and blurring of the peri-esophageal fat space ( Fig. 1 ). Standard gastroscope could not be passed through the esophageal entrance because of significant pharyngeal stenosis and edema, and pus oozing was seen at the entrance of the esophagus ( Fig. 2 ). Pharyngeal infection was considered. After intravenous antibiotics ( Fig. 3 ), infection was controlled but dysphagia remained without any sign of relief. On day 50, an ultra-slim gastroscope revealed three mucosal defects with a diameter of 4–10 mm in the esophagus 18–23 cm from the incisors. The endoscope was entered through the largest mucosal defect into the submucosal layer and white thin pus was found within several submucosal cavities, which formed after self-absorption of pus and indicated phlegmonous esophagitis ( Video 1 ). Enteral nutrition is performed through an endoscopic indwelling gastric tube. On day 55, chest CT showed a significant reduction of esophageal wall thickening ( Fig. 4 ). On day 63, repeat gastroscopy showed two of the three previous mucosal defects healed and one remained there with a size of 5 mm, which was closed by two metal clips. The patient then started eating orally and was discharged on day 70. Phlegmonous esophagitis is rare and there is no standard treatment for phlegmonous esophagitis 1 . Available treatment options include infection control with antibiotics, endoscopic incision 2 3 4 5 , or surgery ( Fig. 5 ). We report the first video of complete access into the abscess cavities of spontaneously ruptured phlegmonous esophagitis, which was achieved with a favorable therapeutic outcome by endoscopic placement of a gastric tube under direct visualization. We believe that gastric tube placement, rather than endoscopic incision or surgery, can result in good outcomes and enable early enteral nutrition in phlegmonous esophagitis with primary spontaneous rupture.

Endoscopy_UCTN_Code_CCL_1AB_2AC

**Fig. 1:**
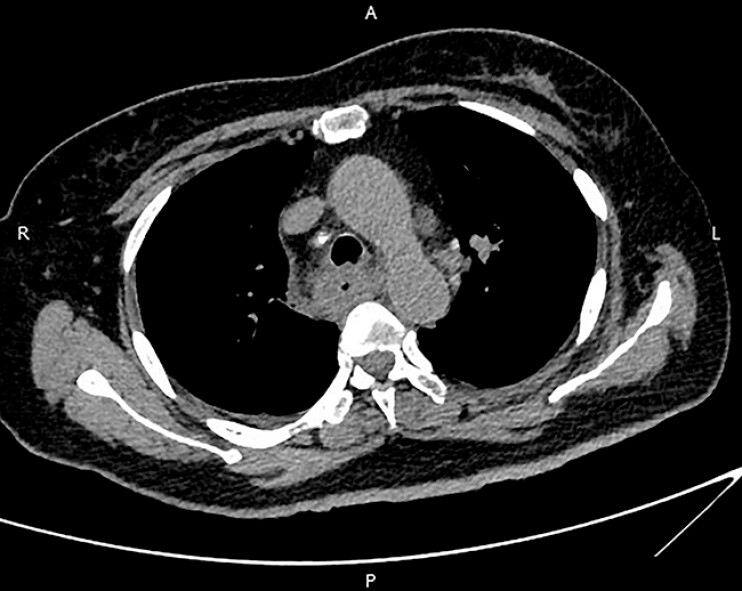
Computed tomography of the chest showed a thickened wall of the entire esophagus and blurring of the peri-esophageal fat space.

**Fig. 2:**
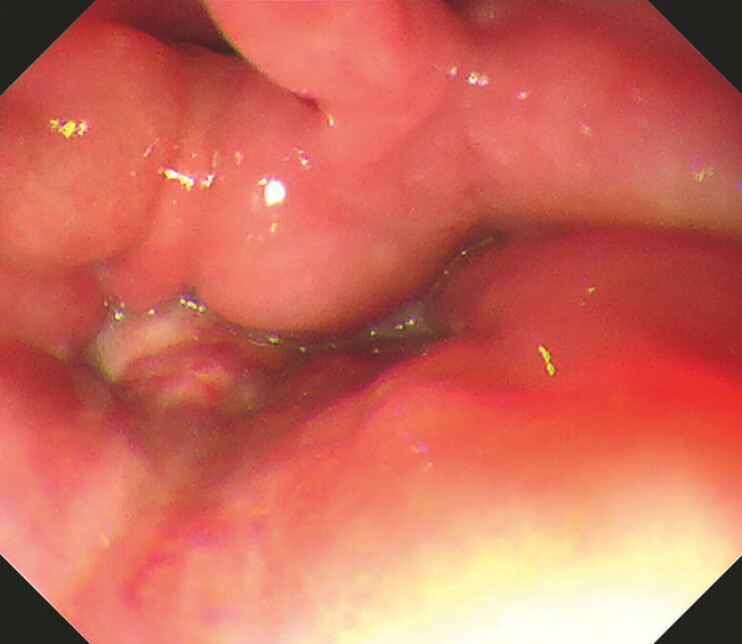
Standard gastroscope could not be passed through the esophageal entrance because of significant pharyngeal stenosis and edema, and pus oozing was seen at the entrance of the esophagus.

**Fig. 3:**
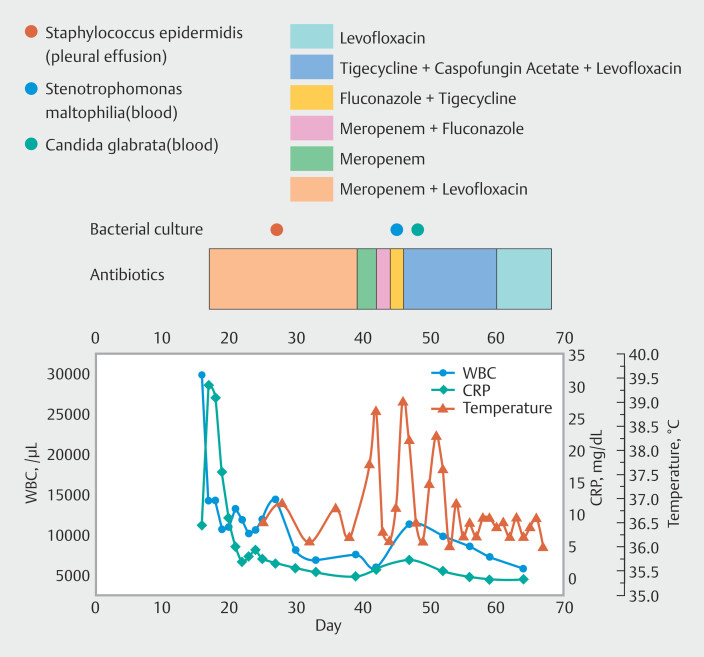
Patient’s bacterial culture results, history of antibiotic therapy, laboratory results, and temperature changes since the patientʼs presentation in the clinic.

**Fig. 4:**
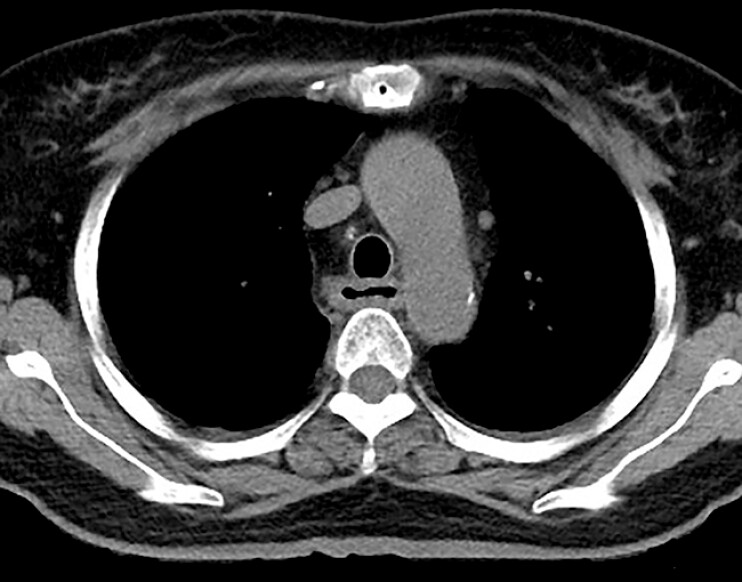
On day 55, computed tomography of the chest showed a significant reduction of esophageal wall thickening.

**Fig. 5:**
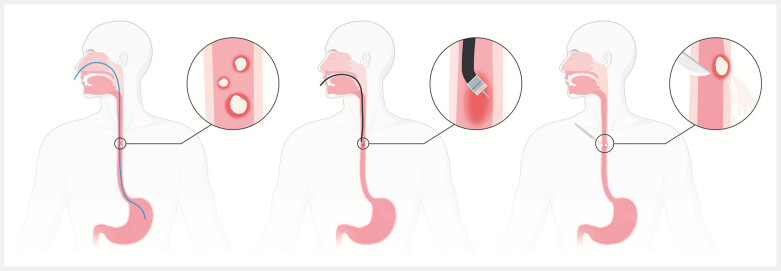
Available treatment options include infection control with antibiotics, endoscopic incision, or surgery. Created in BioRender. Li, G. (2025) https://BioRender.com/t39f227 . [rerif].

The application of ultra-slim gastroscope in the diagnosis and treatment of phlegmonous esophagitis.Video 1

## Notes

Endoscopy E-Videos https://eref.thieme.de/e-videos
*E-Videos* is an open access online section of the journal *Endoscopy* , reporting on interesting cases and new techniques in gastroenterological endoscopy. All papers include a high-quality video and are published with a Creative Commons CC-BY license. Endoscopy E-Videos qualify for HINARI discounts and waivers and eligibility is automatically checked during the submission process. We grant 100% waivers to articles whose corresponding authors are based in Group A countries and 50% waivers to those who are based in Group B countries as classified by Research4Life (see: https://www.research4life.org/access/eligibility/ ). This section has its own submission website at https://mc.manuscriptcentral.com/e-videos .
